# Chilled storage of Pacific white shrimp (*Litopenaeus vannamei*) spermatophores for assisted insemination

**DOI:** 10.1590/1984-3143-AR2024-0006

**Published:** 2024-10-25

**Authors:** Francisco Hiago Gadelha Moreira, Larissa Teixeira Nunes, Vanessa Alves Pereira, Renata Vieira do Nascimento, Carminda Sandra Brito Salmito Vanderley

**Affiliations:** 1 Programa de Pós-graduação em Engenharia de Pesca, Universidade Federal do Ceará, Fortaleza, CE, Brasil; 2 Programa de Pós-graduação em Ciências Veterinárias, Universidade Estadual do Ceará, Fortaleza, CE, Brasil

**Keywords:** spermatophore cooling, powdered coconut water, shrimp reproduction, mineral oil

## Abstract

The cooling of *Litopenaeus vannamei* shrimp spermatophores for assisted insemination can enable the transfer of gametes between reproduction laboratories. This study aimed to assess three extenders for cooling *L. vannamei* spermatophores for assisted insemination. Spermatophores were chilled at 15 °C for 24 or 48 hours using powdered coconut water ACP® (PCW), mineral oil (MO), and sterilized seawater (SSW) as extenders. All treatments demonstrated consistent responses over time. Apparent viability and morphological integrity percentages remained above 60% and 70%, respectively, across treatments and storage durations. Focusing on diluents, normal cell percentages for MO, SSW, and PCW treatments were 74.9±9.20%, 77.3±9.40%, and 78.1±6.35%, respectively, irrespective of storage time. The highest hatching rate was observed in the SSW treatment (80.67±12.01%), which was significantly superior to the PCW treatment (50.15±20.75%). The hatching rates observed in the MO treatment (71.47±18.83%) did not statistically differ from either PCW or SSW treatments. The cooling protocol successfully preserved the spermatophores' ability to maintain favorable levels of apparent viability, normal morphology, and hatching rates after 48 hours of storage at 15 °C using mineral oil, seawater, or ACP® as extenders. Sterilized seawater emerged as the most efficient diluent, delivering superior hatching rates following artificial insemination.

## Introduction

The Pacific White Shrimp *Litopenaeus vannamei* accounted for 51.77% of crustacean aquaculture production in 2020, with a total production of 5,812.2 thousand tons, representing a 52.8% improvement over the past 5 years (FAO, 2022). In Brazil, *L. vannamei* production reached 78.6 thousand tons in 2021, a 18.1% increase compared to the previous year (IBGE, 2022), making Brazil the second-largest shrimp producer in the Americas. Shrimp production in Brazil is primarily concentrated in the Northeast region, where approximately 99% of all cultivated shrimp is produced, and there are expectations for further production growth in the coming years. Despite these advancements, there are still challenges in the management and regulation of the sector. However, significant innovations have been observed in the production chain, such as genetic enhancement, advancements in biotechnology, and the adoption of new technologies (Tahim et al., 2019).

Selective breeding programs have yielded significant results in improving the productive performance of *Litopenaeus vannamei*, particularly in terms of growth and survival ratios (Sui et al., 2016). A successful short-term storage protocol would enhance breeding programs (Nimrat and Vuthiphandchai, 2008) and enable the transportation of gametes (Contreras et al., 2020), facilitating hatchery management and allowing for the exchange of genetic material between different hatcheries. This would also allow the utilization of genetically improved strains without the need to transport live animals. Importation legislation for live breeding of exogenous animals is stringent in some countries, including Brazil, to prevent the introduction and spread of diseases. Therefore, short-term storage of gametes could significantly leverage advancements and innovations in Brazilian shrimp farming by accelerating genetic enhancement through the introduction of improved genetic material from other countries.

Chilled storage aims to preserve sperm for days, weeks, or even months (Gallego and Asturiano, 2019). To achieve this, temperatures above the freezing point are used (Bobbe and Labbé, 2009), and temperature reduction can be accomplished by placing the samples in Styrofoam boxes containing ice (Morales-Ueno et al., 2016) or using automatic refrigerators (Bray and Lawrence, 1998). The use of an appropriate extender medium is necessary to buffer deleterious pH variations, provide substrates to sustain cell metabolism, prevent sperm activation, and enable cellular homeostasis (Bobbe and Labbé, 2009).

Chilled storage protocols for penaeids have been evaluated for *Penaeus monodon* (Feng et al., 2018), *Fenneropenaeus merguiensis* (Nimrat et al., 2020), and *L. vannamei* (Bray and Lawrence, 1998; Nimrat et al., 2006; Morales-Ueno et al., 2016; Castelo-Branco et al., 2016). These studies mainly employed mineral oil, Ringer's solution, and seawater as extenders. However, there are few studies compared to other aquatic animals, leaving room for testing different extenders, temperatures, and methods, such as powdered coconut water (ACP^Ⓡ^), which is a notable extender in fish sperm cryopreservation (Almeida-Monteiro et al., 2020), but has not been reported in shrimp spermatophore assays.

Additionally, there are no reports on the chilled storage of *L. vannamei* spermatophores combined with assisted insemination. However, a vitrification protocol for *L. vannamei* spermatophores has been reported (Castelo-Branco et al., 2015), resulting in high rates of apparent viability after thawing but zero hatching rates after assisted insemination. This indicates that evaluating viability based solely on morphology or apparent viability does not guarantee that spermatophores will be capable of fertilizing eggs and developing into nauplii.

Previous work has primarily focused on in vitro analyses, particularly membrane integrity (apparent viability), since penaeid shrimp sperm cells are non-motile, which precludes motility evaluations commonly performed in fish sperm assays (Cabrita et al., 2014; Salmito-Vanderley et al., 2012). However, it is crucial to evaluate chilled storage protocols through artificial insemination, as this is the primary application for preserved spermatophores. Therefore, the objective of this study was to evaluate different extenders for the cooling of *L. vannamei* spermatophores for the purpose of assisted insemination.

## Methods

### Broodstock management

The shrimp used in this study were provided by a partner shrimp post-larvae production company located in Ceará, Brazil. The broodstock were maintained inside the maturation sector of the partner company and under hatchery routine of management and conditions, including a pH of 8.0, salinity of 36 g L^-1^, temperature of 28 °C, and a photoperiod of 12:12. They were housed in 15 m^3^ tanks filled with treated seawater and fed a diet consisting of squid, mussel, and commercial feed six times a day. All broodstock handling follows the actual hatchery routine. Also, spermatophores collections and assisted inseminations were performed in the same way it is usually performed in the partner company and inside its facilities. After the collection of spermatophores and spawns, the male and female shrimp were returned to the hatchery tanks. Only spermatophore samples and histological slides were taken to the State University of Ceara for analysis. This work was performed using an invertebrate specie and inside of a private company facility and daily management.

### Spermatophore collection

Non-melanized spermatophores were collected from 36 sexually mature male shrimp by applying light manual pressure at the terminal ampoules. The spermatophore on the right side of each male was designated for assisted insemination after the chilled storage period, while the one on the left side was used for sperm quality analysis following the same cooling time.

### Chilled storage

Previous tests were conducted to assess chilled storage conditions for shrimp spermatophores at both 15 °C and 4 °C. Superior results in terms of apparent viability were observed at 15 °C. Immediately upon collection, the spermatophores were transferred to 2 mL cryotubes (Eppendorf) containing 1.2 mL of one of the following cooling diluents: powdered coconut water ACP^Ⓡ^ (800 mOsm/L), filtered sterilized seawater (autoclaved at 127 °C for 90 minutes), and mineral oil (light oil, BioReagent, Sigma-Aldrich). The cryotubes were then placed in a Styrofoam box filled with ice. The amount of ice was adjusted to maintain a temperature of approximately 15°C, and a digital thermometer was used to monitor the temperature inside the Styrofoam box throughout the entire storage period.

Three treatments, each with five replications, were used: MO treatment (mineral oil), PCW treatment (powdered coconut water ACP^Ⓡ^), and SSW treatment (sterilized seawater as diluent). Sperm quality assessment and assisted insemination were performed after 24 and 48 hours of chilled storage. The control group consisted of fresh spermatophores collected and immediately used for sperm quality analysis and assisted insemination.

### Sperm quality analysis

Sperm membrane integrity and morphology were evaluated. For this, a spermatic suspension was prepared by either using freshly collected spermatophores or removing chilled spermatophores from the cryotubes. The spermatophores were placed on a petri dish with the aid of forceps and cut into small pieces (4 or 5 pieces measuring approximately 2 × 2 mm each) with a scalpel blade. Subsequently, all spermatophore pieces from each cryotube were transferred to a new cryotube containing 500 μL of sterilized seawater and manually agitated to release the sperm cells into the water.

Membrane integrity, also known as sperm viability, was analyzed following a modified methodology described by Jeyalectumie and Subramoniam (1989). For this analysis, 10 μL of the spermatic suspension was mixed with 10 μL of eosin and 5 μL of nigrosine. After a 10-minute incubation period, 5 μL of the stained sample was placed between a slide and coverslip for observation under an optical microscope (400x magnification, Opton Microscope; Tucsen, China). A total of 100 sperm cells per slide were counted, with pink cells indicating ruptured membranes and unstained cells against the dark background indicating intact membranes. Sperm viability, represented as the percentage of intact cells, was calculated based on five replicates, totaling 500 cells per treatment. Membrane integrity serves as a robust indicator of sperm viability, and it was determined as the mean across all replicates within each treatment.

The morphological evaluation of sperm cells was performed by adapting the staining and fixation methodology described by Miliorini et al. (2011). Spermatic suspensions were fixed in a 4% formalin solution (1:10; sperm solution: fixative), and 50 μL aliquots were stained with 3 μL of rose Bengal. After a 10-minute rest period for stain interaction with the sperm cells, stained and fixed spermatic solutions were placed between a slide and coverslip for assessment under an optical microscope (400x magnification). Sperm cells with acrosomal spikes and regular head shapes (e.g., oval, pear, round) were considered normal, while those lacking spikes or exhibiting irregular head shapes were deemed abnormal. The results of the morphology analysis were expressed as the percentage of normal sperm. A total of 500 cells per treatment were counted.

### Assisted insemination

Assisted insemination assays were performed by adapting the methodology described by Arce et al. (2000). In brief, each spermatophore designated for insemination post-chilled storage or fresh spermatophores was manually pressed to release the sperm mass and placed over the thelycum of mature females. One technician held the female with both hands, opening the pereopods to expose the thelycum, while another technician deposited the sperm mass onto the thelycum using forceps. One spermatophore was used to inseminate each female. After insemination, the females were transferred to individual spawn tanks (100 L) filled with treated seawater and were continuously monitored. Two assisted insemination assays were conducted, one after 24 hours and another after 48 hours of chilled storage. Each storage time utilized 18 sexually mature females, with five females assigned to each treatment (MO, PCW, and SSW), and three females used for insemination with fresh spermatophores. This resulted in a total of 36 female shrimp used in both assays.

In the first assisted insemination assay (using 24-hour stored spermatophores), 14 females spawned: four from each treatment and two from the fresh spermatophores group. In the second insemination assay, 15 females spawned: five from the SSW treatment, four from both the MO and PCW treatments, and two from the fresh spermatophores group. Approximately 5 hours after assisted insemination, all females were removed, and the eggs were concentrated by filtering them with containers equipped with mesh filters to retain the eggs while allowing water to pass through. Treated seawater was used to wash the eggs and remove any impurities present in the spawning tanks. After this procedure, the eggs from the spawning females were placed in 14 L hatching containers, with each female's eggs kept separate. The hatching containers were equipped with activated aeration to ensure constant oxygenation and homogenization of the eggs. The water used in the spawning tanks and hatching containers had the same pH, salinity, and temperature as the water in the maturation sector.

To calculate the total number of eggs after spawning, two 5 mL aliquots were taken from each hatching container. The eggs were counted visually in petri dishes placed on a dark background. If the two counts differed by more than 10%, the samples were returned to the container, and new samples were taken. The arithmetic mean of the two counts was used to estimate the total number of eggs in each hatching container by multiplying the average number of eggs in 5 mL by the total volume of the container (14 L).

Approximately 16 hours after insemination, hatching was observed, and the nauplii were counted to determine the hatching rate using the same counting protocol as for the estimation of total eggs. However, for the hatching rate, the number of nauplii was counted. Once the number of nauplii was estimated in each tank, the hatching rate was calculated based on the previously counted number of eggs using the Formula 1:


$$Hatching\;rate\;=\;\left(total\;nauplii\;amount\;÷\;total\;eggs\;amount\right)\;x\;100$$ (1)

### Statistical analysis

The data were evaluated using the Shapiro-Wilk test to investigate the normal distribution of residuals and homoscedasticity. After confirming these parameters, the data were subjected to analysis of variance (ANOVA) using the SAS PROC GLM software (2002). The analysis followed a completely randomized experimental design in a 3 × 2 factorial scheme (diluents × storage time). When a statistical difference was observed, a Duncan test was applied to compare the means. The data were expressed as mean ± standard deviation of means (SD) at a significance level of 5% (*P* < 0.05).

## Results

**Figure 1:**
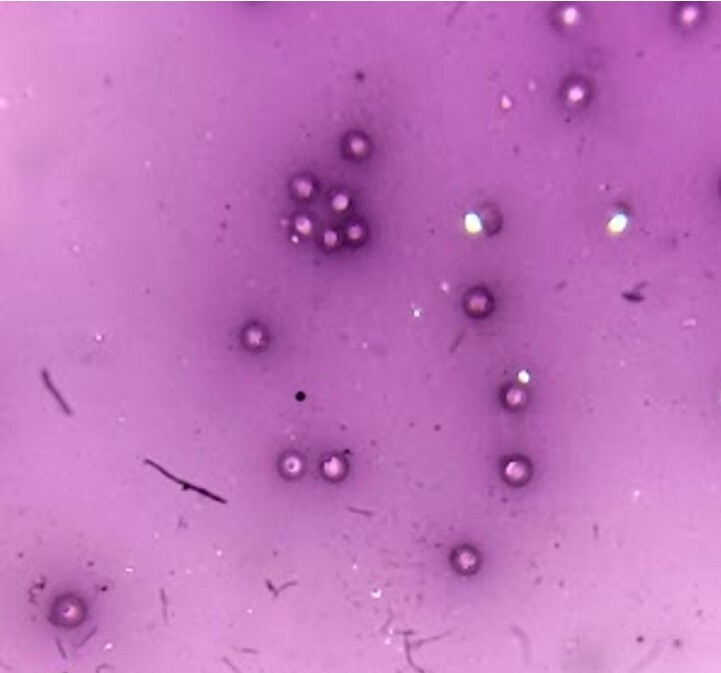
Photomicrograph of *L. vannamei* sperm cells stained with eosin and nigrosine for membrane integrity analysis (adapted from Jeyalectumie and Subramoniam, 1989) under an optical microscope (400x magnification, Opton Microscope; Tucsen, China). Translucent cells, which were not penetrated by eosin stain, were counted as viable sperm cells, while pink cells were counted as non-viable.

All the spermatophores used in this work contained viable spermatozoa capable of carrying out fertilization. The sperm viability, expressed as the percentage of cells not penetrated by eosin (Figure 1), of fresh spermatophores was 73.75 ± 7.72% and the hatching rate of eggs from females inseminated with fresh spermatophores was 72.07 ± 19.28%.

Statistical analysis revealed no interaction between storage time and diluents, indicating that these two variables are independent and cannot be compared.

When all data from treatments were grouped and only storage times were compared, there were no significant differences in sperm viability and the percentage of normal cells after 24 and 48 hours of chilled storage (Table 1; *P* > 0.05). However, the hatching rate significantly decreased from 78.29 ± 14.42% to 57.71 ± 22.01% after 24 and 48 hours of chilled storage, respectively.

**Table 1:** Sperm quality and hatching rate of *Litopenaeus vannamei* spermatophores after 24 and 48 hours of chilled storage. Data from treatments [mineral oil (MO), powdered coconut water (PCW) and sterilized seawater (SSW)] were grouped.

**Parameters**	**Chilled storage time**	
	**24h**	**48h**
Morphology (%)	76.53 ± 6.24^a^	77 ± 10.11^a^
Sperm viability (%)	64.53 ± 13.96^a^	62.30 ± 15.05^a^
Hatching rate (%)	78.29 ± 14.42^a^	57.71 ± 22.01^b^

Statistical significance at *P* <0.05. Different letters indicate a difference between columns (*P* < 0.05). Data are presented as the mean ± standard deviation (SD).

**Figure 2:**
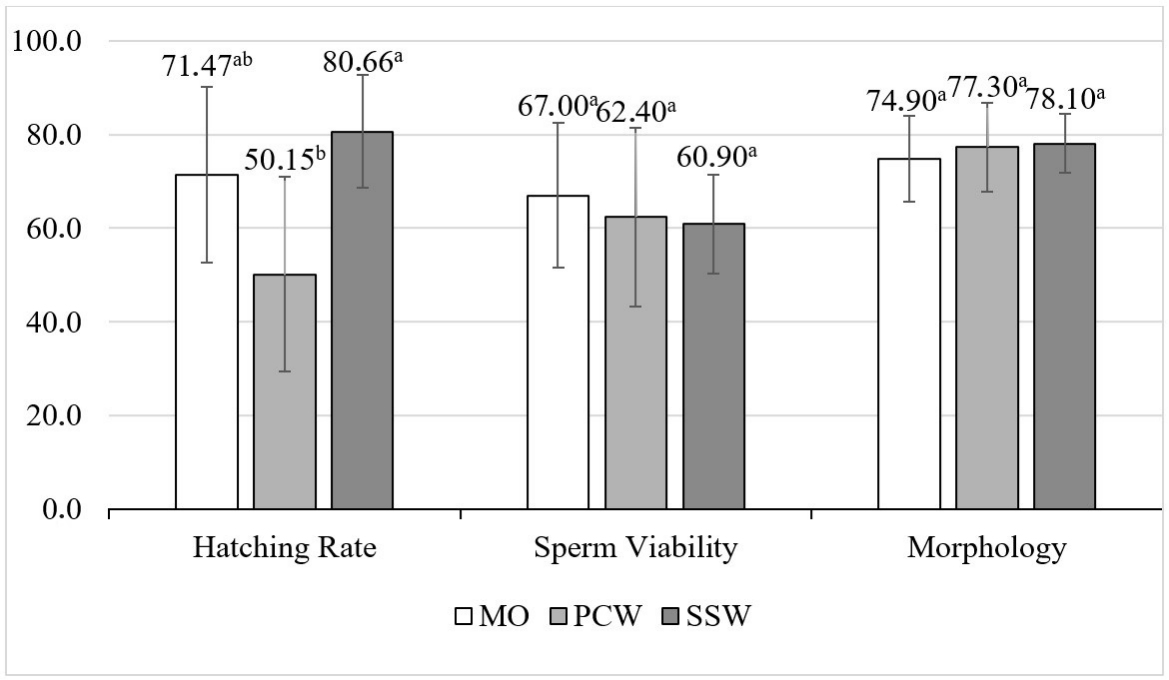
Sperm quality and hatching rate of *Litopenaeus vannamei* spermatophores after chilled storage with three diluents: mineral oil (MO), powdered coconut water (PCW), and sterilized seawater (SSW). Data from both storage times were grouped. Different letters indicate a significant difference between columns (*P* < 0.05). Statistical significance at *P* <0.05.

**Figure 3:**
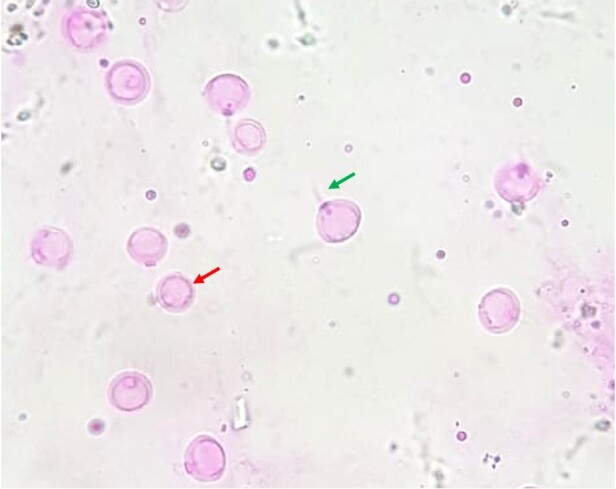
Photomicrograph of *L. vannamei* sperm cells stained with rose Bengal for morphology evaluation (adapted from Miliorini et al., 2011) observed under an optical microscope (400x magnification, Opton Microscope; Tucsen, China). Cells with acrosomal spike were counted as morphologically normal (green arrow). The absence of acrosomal spike (red arrow) or deformed head shapes were considered as abnormalities.

**Figure 4:**
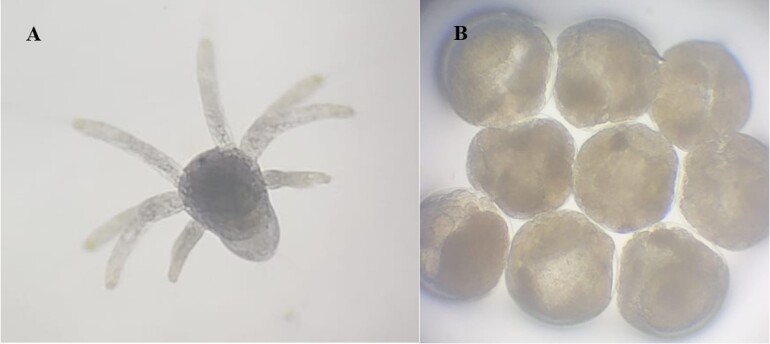
Recently hatched nauplii after assisted insemination and unhatched eggs observed an under optical microscope (400x magnification, Opton Microscope; Tucsen, China). (A) Recently hatched nauplii after assisted insemination; (B) Unhatched eggs after assisted insemination.

When data related to each chilled storage time were grouped and only the three treatments were compared (Figure 2), the percentage of morphologically normal sperm cells was similar in all diluents tested (*P* > 0.05), remaining above 70% of normal cells, which exhibit acrosomal spikes as shown in Figure 3. Furthermore, the means of sperm viability remained above 60% and did not show significant differences (*P* > 0.05). Although the hatching rate results from the SSW treatment were superior to those from the PCW treatment (*P* < 0.05), the results obtained from the MO treatment did not differ significantly from those of both SSW and PCW for the number of hatched nauplii post assisted insemination as shown in Figure 4 (Hatching rate; *P* < 0.05).

## Discussion

All treatments demonstrated similar ability in preserving normal sperm morphology after 48 hours of storage at 15 °C. This finding is consistent with the results reported by Bray and Lawrence (1998) for a shorter storage duration of 36 hours at 15 °C in seawater, where the mean percentage of normal sperm was 77.2%. In contrast, Morales-Ueno et al. (2016) observed high rates of non-viable sperm cells after 36 hours of storage at 14 °C in a saline solution, which differs from the results of the present study. Our study showed a higher percentage of viable cells after a longer cooling period at a similar temperature, suggesting the suitability of the evaluated protocol.

Nimrat et al. (2006) achieved a sperm viability rate of 55.7% for *L. vannamei* spermatophore storage at 2-4 °C using mineral oil over 35 days. However, in the case of *Fenneropenaeus merguiensis*, Nimrat et al. (2020, 2022) found a similar sperm viability rate to our study when using mineral oil for chilled storage for 28 to 31 days. These findings suggest that mineral oil may be effective in prolonging the cooling storage time of spermatophores. Therefore, future studies could explore longer storage times for *L. vannamei* spermatophores using mineral oil.

Mineral oil, composed of refined liquid hydrocarbons and derived from petroleum, possesses properties that contribute to maintaining membrane integrity, normal morphology, and hatching rates. Wale and Gardner (2016) stated that mineral oil reduces pH and temperature fluctuations, delays evaporation, and stabilizes osmolarity when used in human embryo culture for *in-vitro* fertilization. These properties may have contributed to the preservation of sperm quality in our study.

Overall, the 24-hour difference between the two cooling storage times did not significantly affect sperm quality results. However, the hatching rates observed in the sterilized seawater (SSW) treatment were superior to the other treatments throughout the entire storage period.

Although the hatching rate in the powdered coconut water ACP^Ⓡ^ (PCW) treatment was the lowest, it was higher than the results reported by Peralta-Martínez et al. (2019), who performed assisted insemination with fresh *L. vannamei* spermatophores and obtained an average hatching rate of 41.1 ± 27.9%. Caballero-Zamora et al. (2015) also reported a lower mean hatching rate of 21.76% compared to the results obtained in the PCW treatment of our study. However, the hatching rate of PCW exhibited a high variability, suggesting that further evaluation is needed regarding chilled storage for assisted insemination using PCW as an extender and the impact of powdered coconut water’s complex composition on the hatching rate. Powdered coconut water possesses a composition similar to its natural form, including carbohydrates, amino acids, vitamins, minerals, saturated fatty acids, growth factors and phytohormones, such as 3-indole acetic acid (Almeida-Monteiro et al., 2020; Carvalho et al., 2006).

Ren et al. (2020) achieved an 84.6 ± 0.23% hatching rate through natural copulation, indicating that the SSW treatment yielded satisfactory results considering the manipulation and cooling stress to which the spermatophores were subjected for up to 48 hours in our study.

We could not find any records of cooling studies on *L. vannamei* spermatophores involving assisted insemination. This could be due to the infrastructure requirements for maintaining females, the additional time required for such studies, and the need for well-trained and experienced technicians to successfully perform assisted insemination. Inadequate assisted insemination may result in no fertilization or dropping of the spermatophore from the thelycum into the water. However, Nimrat et al. (2007) stored *P. monodon* spermatophores in mineral oil at 2-4 °C for 7-8 days and achieved a hatching rate of 87.6 ± 1.2%. This indicates the possibility of obtaining favorable results even with storage times longer than those evaluated in our study for *L. vannamei* spermatophores. Nevertheless, it is important to highlight the significance of this pioneering work, as it enables the preservation of *L. vannamei* genetic material and facilitates the exchange of genetic material between breeding sites through assisted insemination. This eliminates the need to transport live animals, which could result in mortality or the spread of diseases among shrimp breeding sites.

## Conclusion

Shrimp spermatophores demonstrated the ability to maintain high rates of membrane integrity, normal morphology, and hatching after 48 hours of storage at 15 °C in mineral oil, sterile seawater, or powdered coconut water. Among the tested diluents, sterile seawater is recommended as the most suitable for cooling spermatophores at 15 °C for up to 48 hours due to its ease of acquisition.

All three diluents proved effective in preserving sperm quality during the 24 and 48-hour storage periods. However, further assisted insemination trials using cooled spermatophores for longer durations are needed to determine the optimal storage time for each diluent, ensuring consistently satisfactory hatching rates. It is worth noting that this study represents the first successful attempt at assisted insemination using cooled *L. vannamei* spermatophores, achieving hatching rates comparable to those obtained with fresh spermatophores. These findings highlight the promising potential of this technique in shrimp breeding practice
